# Pennsylvania Hospital

**Published:** 1839-01-26

**Authors:** 


### CLINICAL LECTURES.

### PENNSYLVANIA HOSPITAL.

#### ON CATARACTS.

*Wednesday, November 14th*—Dr. T. Harris remarked: I propose to-day, gentlemen, to operate upon a case of cataract. This term is given to every opacity situated in the crystalline lens, or its capsule. It is usually slow in its formation, and in its early stage is liable to be confounded with amaurosis. Commonly, the opacity is first discerned in the centre of the lens. The patient is first sensible of the impediment to his vision, by seeing objects in a misty or smoky atmosphere, and this symptom generally shows itself before there is any actual opacity to be observed through the pupil. Objects which are placed directly in front of the eye, are seen in the forming stage with greater difficulty than those which are placed obliquely; for the central point behind the pupil being first affected, the direct rays of light are intercepted, while those which fall obliquely are not so obstructed, particularly when the light is not too powerful, and the pupil consequently well dilated. Hence, cataractous patients see better in twilight than in the glare of noon-day. In this stage, too, vision is improved by convex glasses. Incipient cataract does not affect the motions of the iris.

Cataract may be distinguished from amaurosis by the latter having its cloudiness at a great depth behind the pupil, and which exhibits rather a greenish or reddish hue, and that the loss of vision is entirely disproportioned to the visible opacity. Glasses are of no assistance to amaurotic patients at any period of the disease.

Cataract has received different names according to its several varieties. The principal are the hard, the caseous, the milky, and the capsular. Other divisions have been made by modern writers, but those which I have enumerated are most worthy of mention. It is of the first importance to distinguish between these varieties, inasmuch as particular operations are adapted to each of them.

In the *hard* cataract, the lens is smaller than in the natural state. There is thus left a considerable space between the lens and pupil, and in twilight, when the pupil is much dilated, the patient can see with tolerable distinctness. Hence, under this form of the disease, temporary relief is obtained by shading the eyes with the hand, or by the application of belladonna. There being no pressure on the iris, as in large cataract, it preserves its usual irritability. The lens is of a horn color, the centre of which is generally more opaque, and firmer than the circumference. All cataracts, occurring in an advanced period of life, are generally hard. There are, it is true, exceptions to this rule, as we sometimes see hard cataracts in children, and there are not wanting cases of the fluid cataract in old persons.

In the *caseous* cataract, the lens is thickened and enlarged, so that the patient enjoys scarcely any vision. The increased volume of the diseased lens presses the iris forward so as to diminish, or nearly to destroy, the anterior chamber. For the same reason, the iris is rendered sluggish in its motions. Neither twilight, nor the application of belladonna, can improve vision, because the pupil cannot be so far dilated as to permit the rays of light to pass to the seat of vision.

The *milky* cataract has not a uniform appearance. It is less transparent below than above. The darker flakes are seen to alter their position, after rubbing the eyes, or by giving to the head a rapid motion. After thus agitating the contents of the capsule, the cataract exhibits a uniform milky aspect. After the eye has rested for a time, it will again show two distinct layers of fluid—the upper of a much less opaque appearance than the lower. In this form of cataract, as in the caseous, the iris is sluggish in its movements, and the anterior chamber of the eye is diminished in size. The congenital cataract is most frequently of this kind.

The last form of cataract which I shall notice, is the *capsular*. It may be seated either in the anterior or posterior capsule, or in both, but most frequently it is found in the anterior. In many instances, both lens and capsule are affected, and then the disease is termed *capsulo-lenticular*. So long as the opacity is confined to the capsule, the cataract exhibits a spotted or mottled appearance. The obscurity does not always commence in the centre, but sometimes begins in the margin of the capsule. If the anterior capsule be alone affected, the cataract will be more convex than usual. If the posterior is opaque, the cataract will be seen deep within the eye, presenting a concave surface. As this form of cataract is generally the result of inflammation in the adjacent parts extending to the capsule, we often observe this membrane, not only adherent to the iris anteriorly, but posteriorly to the tunica hyaloidea.

The ordinary cause of cataract are still imperfectly understood. In general, however, it is supposed to be the result of inflammation in the lens or capsule. Waller has been able to demonstrate, by means of a powerful microscope, that minute blood vessels may be seen in the substance of the lens itself. He was able to observe these vessels only when the lens and capsule were diseased, and not in a healthy condition of the eye. The vessels, however, must have existed anterior to the disease, and only rendered apparent, as in other white tissues, by the supervention of inflammation. Hence it is supposed that inflammation of the capsule generally precedes opacity of the lens. If, then, the lens, like other parts of the body, is nourished by blood vessels, we are bound to infer that it is capable of the same diseased actions, and be as readily inflamed as other structures.

Cataracts are sometimes produced by metastasis from distant organs. Instances are recorded where it has been caused by repelled itch, gout, suppressed menses, hæmorrhoids, &c. There is nothing more extraordinary observed in this case, than is seen when the eruption of scarlatina is suddenly transferred to the brain.

Before the surgeon attempts to operate for cataract, there are certain circumstances which must be duly considered, which may materially influence the result. For example, when the formation of cataract has been preceded by severe neuralgic pains in the head—where the patient is subject to severe rheumatic pains of the joints—where there exists a tendency to erysipelas of the face—where the patient is subject to attacks of epilepsy—where he has suffered from frequent and severe ophthalmia—where some other local affection, such as amaurosis, glaucome, &c., are complicated with cataract; then the prognosis should be considered unfavourable.

When, on the contrary, the eyes, and the system generally, are in a healthy condition—when there exists none of the complications which I have stated, then the operation may be performed with every reasonable hope of success. Under the most favourable circumstances, we cannot expect uniform success, as untoward incidents may occur to defeat the best performed operations.

It was the practice of the ancients to select the spring of the year as the best season for the performance of operations. This is not the custom of modern surgeons. It is now thought that from the frequent and sudden vicissitudes of the temperature, the patients are much more liable to various acute attacks, than in warm and cold seasons.

It was formerly the practice, before performing operations, to prepare the patient, by bleeding, purging, and diet. We now consider this practice an injurious one; for while it weakens the system, it renders the patient more irritable, and thus makes him more susceptible to the inflammation, which it was intended to prevent.

The operations performed for removing the diseased lens from the axes of vision, consist of extraction, *couching*, or depression, and the *breaking up of the lens.*

These operations have all been successfully performed, but the discriminating and experienced surgeon will select that method which is best adapted to the case before him. No single operation can be adapted to every variety of cataract. It would be folly to attempt to extract a fluid cataract, to cut up a hard one, or to depress a large one.

The rule, then, should be, to either extract or couch a hard cataract, and to cut up those which are milky or caseous.

The last operation is the one which I propose to perform this morning, because the cataract is a soft one. This method is sometimes named keratonyxis, but more frequently Saunders' operation. In performing this operation, surgeons sometimes use the narrow-bladed knife, invented by Saunders, or the straight spear-shaped needle used by Beer. The latter instrument I propose to use in the case which I shall presently introduce. It penetrates the tunics with more facility, and its point may be directed with more precision.

This operation may be performed either by passing the needle through the cornea or sclerotica; the one is called the anterior, the other the posterior operation. As the cornea is less abundantly supplied with blood-vessels and nerves than the sclerotica, it consequently possesses less sensibility, and is less liable to pain and inflammation. In order to give the surgeon sufficient space to enable him to lacerate the capsule, and cut up the lens, the iris should be previously dilated by freely rubbing over the eyebrows and lids the extract of belladonna or stramonium, softened by the addition of a little water. This application should be made three or four hours previously to the operation, so as to give full time to accomplish a complete dilatation. The patient should be placed on a low chair, with the light falling obliquely on the eyes, the head supported against the breast of an assistant, who, at the same time, elevates the eyelid with his fore and middle fingers. The surgeon should be seated on a more elevated chair,—the lower lid of the patient should be depressed by the two fingers of his left hand, while he passes the needle through the cornea about a line anterior to its junction with the sclerotica. Having reached the centre of the pupil, the needle is plunged through it, and the operation completed by dividing the lens into fragments, and forcing a portion of them into the anterior chamber, where they are more or less rapidly absorbed. This operation sometimes requires to be repeated. The most successful cases, indeed, have been those in which I have been obliged to disturb the fragments three or four times.

As there seems to be something peculiar in the case of cataract on which I am about to operate, I will occupy your attention for a few minutes in giving you a history of it. The patient is a female black, aged fourteen years. She was received into the hospital in August last, affected with cataract of both eyes. Eleven months previously to this period the operation of keratonyxis was performed on both eyes, by a surgeon of this city. He thinks he tore the capsules freely. The operator informs me that very little inflammation supervened, and in ten days the cataract appeared as solid and entire as before the operation. When she arrived in the hospital, Dr. Norris, who was then on duty, had not been told that any previous operation had been performed; and, certainly, the appearance of the cataract afforded no evidence of it. The opaque lens seemed perfectly round and entire. A few days afterwards, Dr. Norris performed Saunders' operation on the left eye, but there seemed to follow no visible absorption; but, to his surprise, he saw a depression in the centre of the right lens which he had not touched; and in ten days absorption had made a hole through it sufficiently large to admit a large pocket probe. During the last week or ten days the absorption has ceased. This case is certainly an extraordinary one. For eleven months after the first operations, both cataracts remained unchanged. A few days after the second operation, absorption commenced rapidly for a time, not in the lens last cut up, but in the one which had not been touched for eleven months.

As absorption has ceased for some days in the right eye, I intend to cut up the remaining portion of the fragments, and force them into the anterior chamber.

[Dr. Harris then operated in the manner already described; and as the cataract was soft, found no difficulty in dividing the lens, and forcing it into the position which he desired. Ten days afterwards he operated on the left eye. The absorption has been complete in both, with the exception of a small portion of the capsule, which has become adherent to the side of the iris. The vision is now excellent.]

PTERYGIUM.

*Wednesday, November 28th.*—I propose, to-day, said Dr. Harris, to perform an operation for the relief of another affection of the eye, *pterygium.* This term is used to denote a triangular membrane, which commences at the internal corner of the eye, and gradually extends itself over the cornea, and thus interfering with the powers of vision. The patient whom I show to you as the subject of this affection, is unable to say how long she has been suffering from it—so gradual is it in its progress. It has now, however, encroached upon the cornea, and you can readily understand what an impediment to sight must be offered by the presence of such a film. Pterygium commonly proceeds from the internal canthus of the eye, though it sometimes arises from the external. It always takes the form of a triangle, the apex of which nearly corresponds with the centre of the pupil. Sometimes there are several pterygia upon the same eye, the points of which are directed towards the centre of the cornea. They are apt to unite,—in which case the whole cornea is covered by a film, and total loss of sight is the consequence. It is rare, however, to meet with more than one pterygium upon the eye.

There are two kinds of pterygium;—one, the fleshy, when the disease appears as a thick, fibrous, tendinous-like mass, over the cornea, and is termed *pterygium crassum;* the other appears as a thin, semi-transparent membrane, and is called *pterygium tenue.*

The old operation for the relief of this affection, consists in dissecting it off. Scarpa proposed to divide it near its origin, and to apply caustic to the divided surfaces, to prevent the vessels from coalescing. The old plan of dissecting off the pterygium, is, I think, objectionable, as it is apt to be followed by inflammation and the effusion of lymph, which leaves a permanent bridle upon the eye, preventing its abduction. Some years ago I performed both operations upon an officer, who was affected with this affection in each eye. I was so much pleased with the result of the operation of Scarpa, that I have since used it, to the entire exclusion of the other. In the one case, the film gradually wasted away; while, in the other, there remained a firm, hard, permanent cicatrix. I shall now go through the operation of Scarpa. [This was accordingly done.]

---

*Treacle as a Dressing for Burns.*—The external use of treacle has been recommended in the first and second stages of burns and scalds. Three things are necessary in its employment: freedom from all extraneous substances, which are frequently met with in it; it must be spread on fine, smooth, bleached calico; and it must be cold.—*Lon. Med. Gaz.*

